# Efficacy of plasma vascular endothelial growth factor in monitoring first-line chemotherapy in patients with advanced non-small cell lung cancer

**DOI:** 10.1186/1471-2407-9-421

**Published:** 2009-12-03

**Authors:** Sachin Kumar, Randeep Guleria, Vikas Singh, Alok C Bharti, Anant Mohan, Bhudev C Das

**Affiliations:** 1 Department of Medicine, All India Institute of Medical Sciences (AIIMS), Ansari Nagar, New Delhi-110029, India; 2 Division of Molecular Oncology, Institute of Cytology and Preventive Oncology, I-7, Sector-39, Noida, Uttar Pardesh-201301, India; 3 Dr. B. R. Ambedkar Centre for Biomedical Research, University of Delhi (North Campus), Delhi-110007, India

## Abstract

**Background:**

Along with the development of new cancer therapeutics, more effective tools for the estimation of response to therapy and prediction of disease progression are required for the better management of inoperable cancer patients.

**Methods:**

We studied 134 newly diagnosed and primarily untreated advanced non-small cell lung cancer patients and 100 controls. Forty two patients received platinum-based chemotherapy. Plasma VEGF levels were quantified in all samples at baseline and also before second and third chemotherapy cycle in 42 patients and correlated with response to therapy as assessed by computed tomography after the third chemotherapy cycle.

**Results:**

We observed that, patients who went into remission had significantly lower baseline VEGF levels before second and third cycles of chemotherapy when compared with patients with no change and progression. Plasma VEGF levels showed a greater decrease from cycle 1 to 2 and from cycle 1 to 3 in patients who showed remission in comparison to those with no change or progression. Plasma VEGF levels before the second cycle detected poor response to therapy with a sensitivity and specificity of 76.9% and 75.0%, respectively (area under the ROC curve = 0.724). Early prediction of disease progression was achieved with a sensitivity and specificity of 71.4% for plasma VEGF before cycle 2 (area under the ROC curve = 0.805). The kinetics of VEGF form cycle 1 to 2 and cycle 1 to 3 also gave significant information for predicting disease progression as well as insufficient therapy response.

**Conclusion:**

Monitoring of plasma VEGF levels during the course of first-line chemotherapy could identify patients who are likely to have insufficient response to therapy and disease progression at an early stage. This may help in individualizing treatment and could lead to better management of the advanced stage lung cancer.

## Background

Lung cancer is one of the commonest neoplasms all over the world with approximately 1.35 million new cases worldwide in 2002 [1]. It is the most devastating cause of cancer-related deaths with more than 1.18 million deaths in 2002 [1]. Survival at 5 years measured by the SEER program in the United States is 15%, the best recorded at the population level. The average 5 year survival in Europe is 10%, not much better than the 8.9% observed in developing countries [1]. The poor outcome is attributable to the fact that almost two-third of cases are diagnosed when loco-regional and/or metastatic extension has already occurred [2]. The only treatment available at this stage is chemotherapy and/or radiotherapy. Although response rates for chemotherapy and radiotherapy are low, several studies have demonstrated moderate beneficial effects concerning survival, time to disease progression, and quality of life when compared with best supportive care [3]. The current staging investigations after cytotoxic therapies, like chemotherapy and/or radiotherapy, involve imaging procedures, like computed tomography (CT) and positron-emission tomography (PET)-CT. However, these procedures can monitor only macroscopic alteration of tumor mass, that too after several cycles of chemotherapy (2-3 cycles of chemotherapy and/or radiotherapy). In addition, they also have a risk of exposure to harmful radiation to the patient. Thus, there is a growing need for a simple tool that can help in evaluating the prognosis, monitor the effect of treatment and most importantly predict response to therapy at an early stage. One potential approach could be the quantification of tumor markers as they take into account the heterogeneity of the tumor mass which contain active, silent, apoptotic and necrotic part and they also represent the activity of the residual tumor disease. Therefore, any change in their levels should reflect the change in the tumor mass due to cytotoxic therapies, which mean that they can potentially provide a sensitive and cost-effective method for the prediction of prognosis of cancer patients. Further, serial assessment of tumor markers during cytotoxic therapies may help in predicting response to therapy at an early stage. This would help in optimizing disease management on an individual basis. In non-small cell lung cancer patients, several tumor markers, like CEA, CYFRA 21-1, and nucleosomes have shown considerable potential for predicting diagnosis, prognosis and therapy monitoring [4-9]. However, their potential in a clinical set-up is yet to be realized.

There is now an increasing recognition of the importance of targeted therapies, especially anti-angiogenesis therapies, in solid tumors to improve the overall survival or progression free survival. The two most common approaches currently being explored are i) antibodies to vascular endothelial growth factor (VEGF) or soluble receptors that inhibit the binding of VEGF to its receptor and ii) tyrosine kinase inhibitors blocking downstream signaling from membrane bound tyrosine kinase receptors. It is therefore becoming essential to study the profile of circulating VEGF during cytotoxic therapies in an effort to evaluate its potential in predicting prognosis, response to therapy, and disease progression. We hypothesized that monitoring VEGF levels during the course of cytotoxic therapy may have the potential to predict the clinical benefits in terms of prognosis and response to therapy in advanced NSCLC patients.

In the present study, we investigated the plasma levels of VEGF in a homogeneous group of patients with newly diagnosed and advanced NSCLC during first-line platinum-based chemotherapy to analyze the utility of VEGF for the prediction of insufficient response to therapy and disease progression. In addition, we looked for any correlation between pre-treatment plasma levels of VEGF with various clinico-pathological parameters in patients with non-small cell lung cancer.

## Methods

### Subjects

We evaluated 134 newly diagnosed and primarily untreated advanced stage lung cancer patients and 100 age-matched controls (patients without malignancy). All subjects were enrolled from Out Patient Department of Medicine, All India Institute of Medical Sciences, New Delhi, India between years 2006-2009. For all patients, a diagnosis of lung cancer was confirmed by the histologic examinations of biopsy and/or cytology specimens obtained during fiberoptic bronchoscopy or with a CT-guided procedure. Pre-treatment evaluation also included evaluation of Eastern Cooperative Oncology Group performance status (ECOG), X-ray and computed tomography (CT) scan of the chest and upper abdomen. If necessary, a CT or magnetic resonance imaging (MRI) scan of the brain, and radionuclide bone scan was performed. All the patients were staged according to the American Thoracic Society TNM classification [10]. Epidemiological data including demographics, family history, risk factors, occupational exposure, and histopathological data was recorded. Forty two (42) of these patients received platinum-based chemotherapy for a minimum of 3 cycles. In these patients, response to therapy was classified according to the WHO guidelines defining "partial remission" (R) as tumor reduction ≥ 50%, "progression" (P) as tumor increase ≥ 25% or appearance of new tumor manifestations, and "no change" (NC; stable disease) as tumor reduction <50% or increase <25% [11].

### Measurement of circulating plasma VEGF

Venous blood was collected in sterile EDTA-coated vials from all subjects at baseline (BV1). Blood was also collected before second (BV2) and third (BV3) cycles of chemotherapy from 42 patients receiving platinum-based chemotherapy. Within one hour, samples were centrifuged (2500 g, 10 min), and the plasma was removed, aliquoted and stored at -80°C until analysis. The VEGF was assayed by commercially available sandwich enzyme-linked immunosorbent assay kits (Calbiochem, Darmstadt, Germany) according to the manufacturer's instructions. The limit of sensitivity of the VEGF assay was 9.0 pg/mL. The coefficient of variation was less than 5.0%. The study was approved by the institute's ethics committee. Informed written consent was obtained from all the subjects.

In the present study, we compared plasma VEGF levels in patients with progressive disease (P) with those having remission and stable disease (R+NC) to predict disease progression (evaluation 1). Also, plasma VEGF levels in patients with remission (R) were compared with those having progressive and stable disease (P+NC) in an effort to predict insufficient response to therapy (evaluation 2).

### Statistical analysis

To test the association of therapy at time of staging investigations after 3^rd ^cycle of chemotherapy with overall survival of the patients, Kaplan-Meier curves and log-rank analyses were established for various response groups.

Concerning the biochemical variables, the baseline values of all markers before the first, second, and third cycles (BV1, BV2, and BV3) and the percentage changes (BV1-2 and BV1-3) were considered for statistical analyses. In a first step, all biomarkers were evaluated on their power to univariately discriminate between (a) patients with progression (P) and non-progression (R + NC; evaluation 1) as well as between (b) patients with remission (R) and non-remission (P + NC; evaluation 2) by Wilcoxon test. To identify the diagnostic biomarker for insufficient therapeutic efficacy and progression, receiver operating characteristic curves and corresponding areas under the curve (AUC) was calculated. In addition, sensitivity and specificity (with 95% confidence interval) was calculated at defined cut-off VEGF levels for detecting insufficient therapeutic efficacy and progression.

Demographic information between cases and controls was compared using unpaired t-test for continuous variables and Chi-square test for categorical variables. The comparison of the different patient groups and the circulating VEGF variables was performed using unpaired t-test. All statistical comparisons for VEGF were performed with logarithmically transformed data as VEGF-values were not distributed normally. Median and interquartile ranges for VEGF is given because of their skewed distributions. p value < 0.05 was considered statistically significant. All statistical analyses were performed using SPSS software program for Windows (SPSS 9.1; STATA Corporation, Texas, 77845, USA).

## Results

### Subject characteristics

The general characteristics of the patients and controls are given in table 1. The median age was 56.5 years (range: 33-85) in non-small cell lung cancer (NSCLC) patients and 56.5 years (range: 35-86) in controls. One hundred and twenty one (90.3%) patients were male, while seventy eight (78.0%) controls were male. Twenty six (19.4%) patients and 33 (33.0%) controls were never smokers. The demographic characteristics of NSCLC patients and controls were significantly different for sex and smoking status. The characteristics of patients with NSCLC, like performance status, histology, stage, tumor size, nodal status, presence of metastasis, smoking and tobacco habits are given in table 2. Briefly, there were 103 patients with squamous cell carcinoma, and 31 with adenocarcinoma. According to TNM classification, 87 patients had stage III disease and 47 patients had stage IV disease.

**Table 1:** Characteristics of the subjects

Characteristics	Cases (n = 134)	Controls (n = 100)
Age (years), median (range)^‡^	56.5 (33-85)	56.5 (35-86)
Sex^#^		
Male	121 (90.3%)	78 (78.0%)
Female	13 (9.7%)	22 (22.0%)
Smoking status^†^		
Never smokers	26 (19.4%)	33 (33.0%)
Smokers	108 (80.6%)	67 (67.0%)
VEGF levels (pg/mL) *	265.7 (155.7-408.0)	74.5 (40.3-132.0)
Median (25-75% quartile)		

^‡^p = 0.642;^# ^p = 0.009;^† ^p = 0.026; * p < 0.0001

**Table 2:** The relationship between clinicopathological factors and plasma VEGF levels

		Plasma VEGF Levels, pg/mL		
		
Characteristics	No. (%)	Median	25-75% Quartile	p Value
Age				
<60	79 (59)	267.4	169.5 - 454.0	0.465
>60	55 (41)	262.7	146.6-385.4	
Sex				
Male	121 (90.3)	264.0	154.0 - 392.6	0.151
Female	13 (9.7)	338.4	262.0 - 459.4	
Smoking				
Smokers	108 (80.6)	273.9	173.7 - 442.7	0.144
Never-smokers	26 (19.4)	257.1	120.7 - 354.5	
ECOG				
1	39 (29.1)	225.7	108.6 - 354.5	0.062
2-3	95 (70.9)	282.0	182.3 - 446.0	
Histology				
SCC	103 (76.9)	276.0	169.5 - 439.5	0.147
ADC	31 (23.1)	262.0	132.3 - 338.4	
Stage				
III	87 (64.9)	251.4	149.0 - 433.4	0.250
IV	47 (35.1)	276.0	214.0 - 399.4	
Tumor Size				
<3 cm	44 (32.8)	172.4	93.0 - 262.0	0.001
>3 cm	90 (67.2)	339.9	218.0 - 484.5	
T Factor				
T2-T3	66 (49.2)	270.9	149.0 - 379.0	0.653
T4	68 (50.8)	263.0	157.6 - 439.7	
N Factor				
N0-N1	65 (48.5)	245.7	121.4 - 360.7	0.082
N2-N3	69 (51.5)	285.7	192.0-439.5	
M Factor				
M0	87 (64.9)	251.4	149.0 - 433.4	0.250
M1	47 (35.1)	276.0	214.0 - 399.4	
Tobacco				
Yes	25 (18.7)	252.3	185.7 - 354.5	0.524
No	109 (81.3)	267.4	154.0 - 433.4	

VEGF, vascular endothelial growth factor; ECOG, Eastern Cooperative Oncology Group; NSCLC, non-small cell lung cancer; SCC, squamous cell carcinoma; ADC, adenocarcinoma; T, tumor; N, node; M, metastasis

### Correlation of patient's characteristics with survival

**Figure 1:**
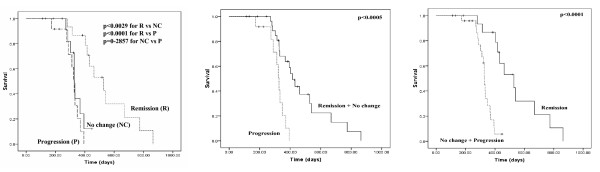
**Correlation of therapy response and overall survival**. 1A, Kaplan-Meier survival curves for overall survival of patients with remission (R), no change (NC), and progression (P) at time of staging investigations after third therapy cycle. 1B, for evaluation 1, patients with progressive disease (P) were compared with patients having remission and stable disease (R + NC). 1C, for evaluation 2, patients with remission (R) were compared with patients having progressive and stable disease (P + NC).

At staging investigations after third cycle of chemotherapy, 16 (38.1%) of the 42 patients had partial remission, 14 (33.3%) showed progression, and 12 (28.6%) had stable disease. The median survival time was 370 days (95% CI = 289.6-450.4). Kaplan-Meier curves for overall survival showed highly significant differences in patients with remission (R), and no change (NC) or progression (P) at time of staging investigations after the third therapy cycle (p < 0.0029 between R and NC; p < 0.0001 between R and P; Figure 1A) with median survival times of 528 (R), 328 (NC), and 327 (P) days, respectively. There was no difference in overall survival time between patients with no change and progression (p = 0.2857). For evaluation 1, when patients with remission and stable disease (R+NC) were compared with patients having progressive disease (P), a significant difference in overall survival was observed with median survival times 416 (R+NC) and 327 (P) days (p < 0.0005; Figure 1B). When patients with remission (R) were compared with patients having progressive and stable disease (P+NC; evaluation 2), median survival times were 528 (R) and 328 (P+NC) days (p < 0.0001; Figure 1C).

We also examined univariate survival analysis for variables including age, stage, T factor, nodal status, performance status, histology, smoking and tobacco habits and plasma VEGF levels (Table 3). Patients with a pre-treatment plasma VEGF levels < 275.2 pg/mL (median value) had a median survival of 348.0 days (95% CI = 246.1-449.9), while those with ≥ 275.2 had a median survival of 370.0 (95% CI = 288.3-451.7). The pre-treatment plasma VEGF levels did not correlate with survival (p = 0.8852). We observed a significant correlation between tobacco habit and survival time (p = 0.022; Table 3). However, no correlation between other prognostic factors with survival was observed (Table 3).

**Table 3:** Univariate survival analysis of prognostic factors of NSCLC (n = 42)

Variables	n	Median survival (95% CI) (days)	P
Age (years)			
<60	27	370.0 (301.3-438.7)	0.665
≥ 60	15	333.0 (244.6-421.4)	
Stage			
III	23	416.0 (369.1-462.8)	0.163
IV	19	327. 0 (298.7-355.3)	
T factor			
T2-T3	17	431.0 (315.3-546.7)	0.051
T4	25	348.0 (248.3-447.6)	
N factor			
N0-N1	21	394.0 (312.2-475.8)	0.380
N2-N3	21	348.0 (310.1-385.9)	
ECOG			
1	30	403.0 (327.9-478.0)	0.130
2-3	12	333.0 (311.5-354.5)	
Histology			
SCC	31	367.0 (288.1-445.9)	0.270
ADC	11	394.0 (324.8-463.1)	
Smoking habit			
Smokers	36	370.0 (294.0-446.0)	0.745
Non-smokers	06	367.0 (248.7-485.3)	
Tobacco habit			
Yes	08	394.0 (352.3-435.6)	0.022
No	34	314.0 (266.0-362.0)	
Plasma VEGF (median)			
<275.2 pg/mL	21	348.0 (246.1-449.9)	0.885
≥ 275.2 pg/mL	21	370.0 (288.3-451.7)	

T, tumor; N, nodal status; ECOG, Eastern Cooperative Oncology Group; VEGF, vascular endothelial growth factor; 95% CI, 95% confidence interval.

### Evaluation of plasma VEGF levels in various response groups

**Figure 2:**
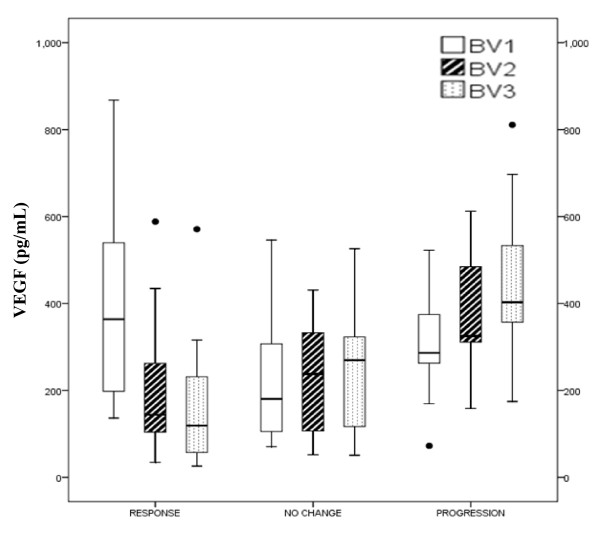
**Distribution of plasma VEGF levels in various response groups**. Median and interquartile range along with minimum and maximum values and outliers of the baseline values of the VEGF before start of therapy cycle 1 (pre-treatment; BV1), cycle 2 (BV2), and cycle 3 (BV3) are given.

We quantified plasma VEGF levels before first (BV1), second (BV2) and third (BV3) cycles of chemotherapy in 42 NSCLC patients and correlated it with response to therapy. Except at cycle 1 (BV1), most patients with remission had considerably lower baseline VEGF levels before the various treatment cycles (BV2 and BV3) than patients with no change and even more than patients with progressive disease (Table 4 and Figure 2).

**Table 4:** Value distribution of VEGF in various response groups of NSCLC patients during first-line chemotherapy

				P	
				
Biomarkers (unit)	Remission median (range)	No Change median (range)	Progression median (range)	R+NC vs P	R vs NC+P
VEGF BV1 (pg/mL)	364.0 (136.6-868.0)	180.5 (70.6-546.0)	286.4 (72.6-522.6)	0.589	0.083
VEGF BV2 (pg/mL)	144.0 (34.6-588.4)	237.8 (52.1-431.0)	325.2 (158.4-612.1)	0.001	0.016
VEGF BV3 (pg/mL)	119.0 (26.0-571.0)	269.7 (51.0-526.0)	402.8 (174.6-811.0)	0.001	0.001
VEGF BV1-2 (Dec%)	41.6 (2.2-86.8)	-6.6 (-90.6-64.5)	-18.5 (-118.2-2.7)	0.0001	0.0001
VEGF BV1-3 (Dec%)	63.0 (17.2-93.1)	-3.2 (-142.2-53.0)	-41.0 (-140.5- -17.8)	0.0001	0.0001
VEGF BV2-3 (Dec%)	12.7 (-62.4-87.1)	-21.2 (-33.6-33.8)	-15.3 (-66.3-5.3)	0.0268	0.0052

BV1, BV2, and BV3, baseline VEGF levels before therapy cycle 1 to 3, respectively; BV1-2, BV1-3 and BV2-3, kinetics of baseline VEGF levels from cycles 1 to 2, 1 to 3 and 2 to 3, respectively; Dec%, decrease in percent (negative numbers meaning increase of the VEGF levels). P values by Wilcoxon test indicate differences between patients with progressive disease (P) and those having remission and stable disease (R+NC; evaluation 1) as well as between patients with remission (R) and those having progressive and stable disease (P+NC; evaluation 2).

When the VEGF levels at BV1, BV2 and BV3 were compared between R+NC versus P (evaluation 1) and R versus NC+P (evaluation 2), it was observed that pre-treatment VEGF levels (BV1) could not predict insufficient response to therapy and disease progression (Table 4). However, baseline VEGF levels before cycle 2 (BV2) and cycle 3 (BV3) were significantly able to predict insufficient response to therapy and disease progression. Additionally, VEGF levels showed stronger decrease from cycle 1 to 2 (BV1-2), from cycle 1 to 3 (BV1-3), and from cycle 2 to 3 (BV2-3) in patients with remission than with no change or progression (Table 4 and Figure 2). The fraction of patients with lower VEGF levels at cycle 2 (BV2) compared with at cycle 1 (BV1) in the various groups were 87.5% (R), 41.7% (NC), and 14.3% (P) and at cycle 3 (BV3) were 100% (R), 50% (NC) and 7.1% (P), respectively.

### VEGF levels during chemotherapy for predicting insufficient response to therapy and disease progression

**Figure 3:**
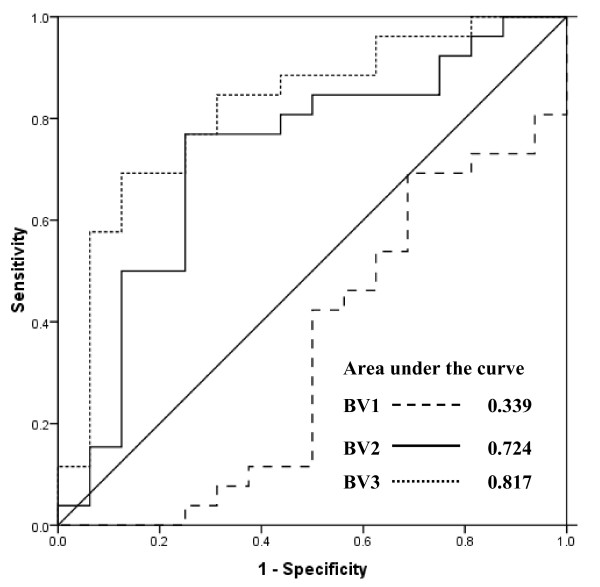
**Receiver-operating characteristics (ROC) curves for an early estimation of response to therapy by plasma VEGF levels available before the start of therapy cycle 1 (BV1), cycle 2 (BV2), and cycle 3 (BV3)**.

To test the potential of VEGF levels as a biomarker for the prediction of insufficient therapy response, we plotted receiver-operating characteristics (ROC) curves for the values available before first (BV1), second (BV2) and third (BV3) therapy cycle (Figure 3). We did not consider plasma VEGF before cycle 1 (BV1) for predicting insufficient therapy response because of very low area under the ROC curve (AUC = 0.339; standard error = 0.09; 95% CI = 0.157-0.520). The cut-off value of plasma VEGF levels before therapy cycle 2 (BV2) was determined as 232.1 pg/mL using the ROC analysis (area under the curve = 0.724; standard error = 0.087; 95% CI = 0.553-0.893) According to this cut-off value, we could predict insufficient therapy response with a sensitivity and specificity of 76.9% (95% CI = 55.9-90.2) and 75.0% (95% CI = 47.4-91.7), respectively (Table 5). Interestingly, we observed similar sensitivity and specificity for plasma VEGF levels available before therapy cycle 3 (BV3; area under the ROC curve = 0.817; standard error = 0.070; 95% CI = 0.679-0.956). For the specific prediction of insufficient response to therapy, we observed very low sensitivities of 3.8% and 11.5% at 100% specificity for the VEGF levels available before second and third cycles of chemotherapy, respectively

**Table 5:** Diagnostic profiles of VEGF levels available before second and third cycles of chemotherapy for the prediction of cancer progression or response to therapy

	AUC	Cut-off	Sensitivity %	Specificity %	LR
	(95% CI)	VEGF (pg/mL)	(95% CI)	(95% CI)	
**Evaluation 1 (R + NC vs P)**					
VEGF BV2	0.805 (0.675-0.935)	312.6	71.4 (42.0-90.4)	71.4 (51.1-86.0)	2.5
VEGF BV3	0.895 (0.797-0.993)	355.4	85.7 (56.2-97.5)	89.3 (70.6-97.2)	8.0
**Evaluation 2 (R vs NC + P)**					
VEGF BV2	0.724 (0.553-0.893)	232.1	76.9 (55.9-90.2)	75.0 (47.4-91.7)	3.1
VEGF BV3	0.817 (0.679-0.956)	261.0	76.9 (55.9-90.2)	75.0 (47.4-91.7)	3.1

BV2, and BV3, baseline VEGF levels before therapy cycle 2 to 3, respectively; AUC, area under receiver operating characteristic

(ROC) curve; 95% CI, 95% confidence interval; LR, likelihood ratio; R, remission; NC, no change; P, progression.

**Figure 4:**
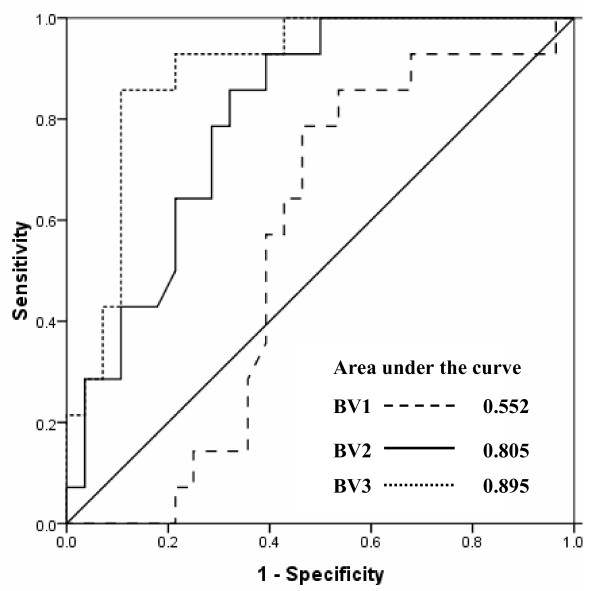
**Receiver-operating characteristics (ROC) curves for predicting disease progression by plasma VEGF levels available before the start of therapy cycle 1 (BV1), cycle 2 (BV2), and cycle 3 (BV3)**.

We also tested the relevance of VEGF levels available before first (BV1), second (BV2), and third (BV3) therapy cycle in predicting disease progression (Figure 4). Because of low value of plasma VEGF before cycle 1 (BV1) under the ROC curve, we did not consider it for predicting disease progression (AUC = 0.552; standard error = 0.08; 95% CI = 0.376-0.729). However, plasma VEGF at BV2 and BV3 most efficiently identified patients with disease progression with area under the ROC curve being 0.805 (standard error = 0.066; 95% CI = 0.675-0.935) and 0.895 (standard error = 0.05; 95% CI = 0.797-0.993), respectively (Figure 4). At a cut-off of 312.6 pg/mL, determined from ROC curve for VEGF at BV2, we observed a sensitivity of 71.4% (95% CI = 42.0-90.4) and specificity of 71.4% (95% CI = 51.1-86.0) for predicting disease progression (Table 5). Further, at 355.4 pg/mL cut-off value for VEGF at BV3, a sensitivity of 85.7% (95% CI = 56.2-97.5) and specificity of 89.3% (95% CI = 70.6-97.2) was achieved (Table 5). For the specific prediction of disease progression, we observed very low sensitivities of 7.1% and 21.4% at 100% specificity for the VEGF levels available before second and third cycles of chemotherapy, respectively.

### Circulating plasma VEGF levels as a tumor marker: calculating specificity and sensitivity using receiver-operating characteristics (ROC) curve

The median plasma VEGF level (25-75^th ^quartile) was 265.7 pg/mL (155.7-408.0 pg/mL) in patients with NSCLC (n = 134) and 74.5 pg/mL (40.3-132.0 pg/mL) in controls (n = 100). This difference in VEGF levels in case versus controls was highly significant (p < 0.0001; Table 1).

**Figure 5:**
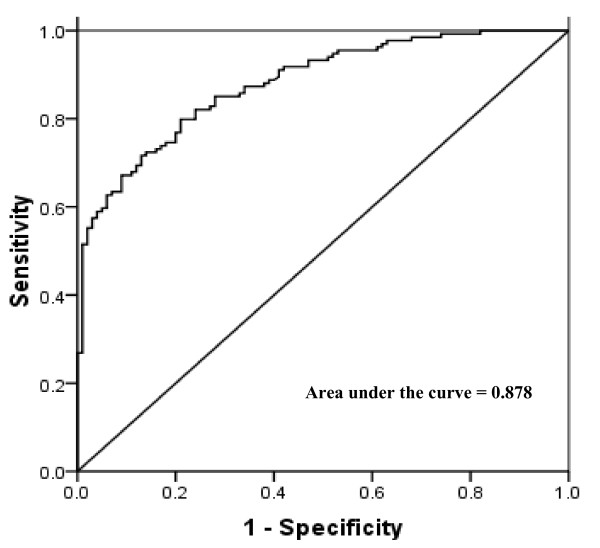
**Receiver-operating characteristics (ROC) curve to calculate sensitivity and specificity of plasma VEGF levels as a tumor marker in advanced non-small cell lung cancer**.

To achieve optimum sensitivity and specificity, a VEGF concentration in patients that did not overlap with the control VEGF value would be ideal. Receiver-operating characteristics (ROC) curve was, therefore, generated for calculating the sensitivity and specificity of VEGF as a tumor marker at a selected cut-off value. The cut-off value of plasma VEGF levels was determined as 145.4 pg/mL using the ROC analysis (area under the curve = 0.8799; standard error = 0.0213; 95% CI = 0.8381-0.9216; Figure 5). At this cut-off, plasma levels of VEGF reached a sensitivity of 79.8% and a specificity of 79.0% for the detection of NSCLC. Further, at 95% specificity, we could detect NSCLC with a sensitivity of 59.7% (VEGF cut-off = 237.0 pg/mL).

### Circulating plasma VEGF levels in correlation with various clinicopathological factors

The relationships between various clinicopathological factors and plasma VEGF levels in the patients with lung cancer are shown in Table 2. VEGF levels were significantly higher in patients with tumor size >3 cm (339.9 pg/mL (218.0-484.5 pg/mL) as compared to patients with tumor size <3 cm (172.4 pg/mL (93.0-262.0); p < 0.001). Plasma VEGF levels were found to be higher in patients with poor performance status (PS), i.e. with a PS 2-3 compared to those with PS of 1 and in patients with higher nodal status (N2-N3) than those with N0-N1. However, these levels did not achieve statistical significance (p = 0.062 for PS and p = 0.082 for nodal status). There was no association between plasma VEGF levels and age, sex, smoking and tobacco habits, histology, and stage (Table 2).

## Discussion

Tumor cells secrete and synthesize a number of angiogenic factors which play an important role in tumor proliferation, spread and metastasis. VEGF, one of the most potent angiogenic factors, plays a central role in the regulation of tumor angiogenesis. The current interest in targeted therapies against VEGF and its receptors has generated the need to study the course of this circulating angiogenic protein molecule during chemotherapy, to find out whether VEGF can be used in predicting prognosis and in monitoring response to therapy. Previously, some tumor biomarkers, including CYFRA 21-1, CEA and nucleosomes [4-9] have been used for predicting prognosis and estimating response to therapy. However, till date, none of them have been validated in a clinical setting. To the best of our knowledge, this is the first study which reports the kinetics of circulating VEGF levels during the course of chemotherapy in an effort to predict insufficient response to therapy and disease progression at an early stage.

In this study, we analyzed the course of circulating plasma VEGF levels at three different time points in 42 advanced stage NSCLC patients receiving platinum-based chemotherapy. VEGF levels were measured before first (BV1), second (BV2) and third (BV3) therapy cycle. These levels were then correlated with available clinical information to know whether VEGF levels could predict insufficient therapy response or disease progression. We found that patients with remission had significantly lower VEGF levels at BV2 and BV3 as compared to patients with no change or progression. However, at BV1, patients who went into remission had higher VEGF levels than those with no change or progression. Further, to correlate any change in VEGF levels with therapeutic response, the kinetics of VEGF levels during the course of chemotherapy was monitored. We observed a stronger decrease in VEGF levels from cycle 1 to 2 (BV1-2), from cycle 1 to 3 (BV1-3), and from cycle 2 to 3 (BV2-3) in patients with remission when compared to those with no change or progression.

Our results with VEGF are in accordance with the hypothesis that, when monitoring the efficacy of cytotoxic therapy, a substantial decrease in the levels of tumor marker often correlates with response to therapy whereas an increase or an insufficient decrease are generally associated with no response or disease progression. In our study, it was noteworthy that a significant difference in VEGF levels was observed in the response group before the start of the second therapy cycle. In a few patients who went into remission, we observed a step fall in VEGF levels to just above the reference range within this short time frame. This decrease in VEGF levels in responders may be due to the effect of cytotoxic agents on tumor cells, either by killing them or by reduction of cellular turnover, leading to a decrease in the number of cells synthesizing and secreting various angiogenic proteins, including VEGF. In this study, it was decided to evaluate VEGF levels after 2 weeks of first and second chemotherapy cycle. At such time points, it could be expected that no significant drug levels would be in circulation and that therefore, VEGF levels will reflect angiogenic activity of the residual tumor mass.

There are few studies which measured serum VEGF levels before and after chemotherapy and the data is conflicting. In a cohort of 29 patients with lung cancer, serum VEGF levels were measured before and after cisplatin-based chemotherapy [12]. A significant decrease in VEGF levels was observed in responders while in non-responders, there was an increase in VEGF levels, an observation similar to ours. In another study, no difference in serum VEGF levels was observed from samples taken before first cycle after 24 hours and 48 hours of cisplatin-based chemotherapy in NSCLC patients [13]. Similarly, in a study of advanced NSCLC patients on cisplatin and gemcitabine regimen, no difference in serum VEGF levels was observed before first and after 3^rd ^cycles of chemotherapy [14]. In a phase II study of stage III NSCLC patients on concurrent chemoradiation given with celecoxib (COX-2 inhibitor), serum VEGF levels did not predict response [15]. Circulating VEGF levels were also used for predicting response in NSCLC patients on second-line chemotherapy. Yoshimoto et al. [16] measured serum VEGF levels before and after second-line gefitinib therapy in 52 NSCLC patients and observed no significant change after the treatment, even in responders. In a multi-centric phase II study of 58 NSCLC patients on second-line treatment, a decrease in serum VEGF levels after two cycles was significantly associated with clinical response [17]. Circulating VEGF levels have also been monitored during the course of chemotherapy in SCLC patients. Tas et al. [18] measured VEGF levels before first cycle and after 2^nd ^cycle of chemotherapy in 34 SCLC patients. There was no significant difference in serum VEGF levels before after cytotoxic therapy (18). In another study, a non-significant increase in serum VEGF levels was observed in both responders and non-responders after 3^rd ^cycle of chemotherapy in a group of 39 SCLC patients [19].

Recently, a number of studies have been done where pre-treatment VEGF levels were correlated with progression-free survival (PFS), overall survival (OS), and response to treatment in lung cancer patients being treated with anti-angiogenesis drugs alone or in combination with conventional chemotherapy. In a recent study by Hanrahan et al [20] pre-treatment VEGF levels were correlated with PFS in three randomized phase II trials in advanced NSCLC. They found that, in NSCLC patients on second/third line treatment, those having low baseline plasma VEGF level had a significantly superior PFS when treated with vandetanib (VEGF receptor/EGFR receptor inhibitor) monotherapy compared with gefitinib monotherapy. Similarly, in a Japanese study, baseline plasma VEGF levels were lower in patients who experienced clinical benefit after vandetanib treatment [21]. Both studies suggest that determining pre-treatment circulating VEGF levels may have the potential to identify patients who would derive benefit from first-line targeted therapy with vandetanib. In contrast to this, in the Eastern Cooperative Oncology Group 4599 trial of carboplatin-paclitaxel with or without bevacizumab in NSCLC, high baseline plasma VEGF were associated with a greater response rate with the use of bevacizumab but not with improved survival [22]. We also observed a greater response rate in patients with higher pre-treatment plasma VEGF levels in NSCLC patients treated with platinum-based chemotherapy.

There are few reports available correlating pre-treatment VEGF levels with response to therapy and overall survival of patients treated by conventional chemotherapy. In a study on 52 advanced stage NSCLC patients treated with cisplatin plus vinorelbine, no correlation between pre-treatment serum VEGF levels and response to therapy was observed [23]. This observation is similar to ours on NSCLC patients treated with platinum doublets. In contrast, Laack et al [24] reported a significant correlation between pre-treatment serum VEGF levels and response to therapy in metastatic NSCLC patients treated in a randomized phase III trial comparing gemcitabine plus vinorelbine (GV) and gemcitabine plus vinorelbine plus cisplatin (GVP). Data regarding association of VEGF levels with survival in NSCLC is conflicting, some studies showing a correlation [25-27] whereas other not being able to do so [15,28,29].

In the present study, we observed a significant difference in overall survival time between patients with remission as compared to patients with no change or progression. During the estimation of therapy response, patients are generally grouped into responders (patients with remission) and non-responders (patients with stable disease or progression). To increase the overall survival of the patients, it is of utmost importance to find the group of patients, at an early stage of their treatment, who are either (i) going to respond to the treatment or (ii) going to progress to a more advanced stage of disease. For both these groups, it would be necessary to focus on the markers already being available before start of the second course of the treatment. This could be helpful in the better management of the cancer patients as an early prediction of response to therapy or disease progression can be used to either intensify treatment or change the treatment plan. Therefore, we conducted two different evaluations of plasma VEGF levels, available before cycle 2 of therapy (BV2), to address both clinical questions.

For evaluation 1 (R+NC versus P), we selected a VEGF (BV2) cut-off levels of 312.6 pg/mL from the ROC curve (area under the curve = 0.805). At this cut-off, we were able to diagnose disease progression with a sensitivity and specificity of 71.4%. While for evaluation 2 (R versus NC+P), VEGF levels before cycle 2 (BV2) was able to predict an insufficient therapy response with a sensitivity and specificity of 76.9% and 75.0%, respectively, when a cutoff of 232.1 pg/mL was applied. Interestingly, the kinetics of VEGF form cycle 1 to 2 (BV1-2) and cycle 1 to 3 (BV1-3) also gave significant information for predicting disease progression as well as insufficient response to therapy.

Although, our sample size was a limitation, the results show that monitoring the levels of circulating VEGF during the course of cytotoxic therapy can predict insufficient response to therapy and disease progression. Earlier studies have not evaluated measurement of levels of VEGF at defined intervals during the initial treatment phase for predicting insufficient response to therapy and disease progression in NSCLC patients. So, a large-scale prospective validation study is needed to confirm the relevance of the current findings presented here.

Further, we compared plasma VEGF levels in 134 NSCLC patients and 100 controls. Also, correlation with various clinico-pathological factors and VEGF was looked at. This study highlights a few significant findings: (a) plasma VEGF levels were significantly higher in patients with NSCLC as compared to controls; (b) significant association was observed between plasma VEGF levels and tumor size. Also, there was some evidence to suggest a correlation between plasma VEGF levels and performance status, and nodal involvement. However, these associations did not reach the level of statistical significance.

## Conclusion

In conclusion, to our knowledge, this is the first study investigating the initial changes in plasma VEGF levels for the prediction of insufficient response to therapy and disease progression in advanced NSCLC patients during first-line chemotherapy. Our results clearly show the relevance of circulating VEGF levels in differentiating responders from non-responders to therapy and in predicting disease progression. In future, such analyses could also be used for monitoring of tumor recurrence.

## Conflicts of interests

The authors declare that they have no competing interests.

## Authors' contributions

SK carried out the experimental work, participated in study design and manuscript preparation. RG participated in study design and manuscript drafting. VS participated in statistical analysis. ACB helped in study design and coordination. AM and BCD helped in study design, statistical analysis, and manuscript editing. All authors read and approved the final manuscript.

## Pre-publication history

The pre-publication history for this paper can be accessed here:

http://www.biomedcentral.com/1471-2407/9/421/prepub
